# Hospital and Health News

**Published:** 1924-03

**Authors:** 


### HOSPITAL AND HEALTH NEWS.

### Next Sanitary Congress.

The thirty-fifth Congress of the Royal Sanitary Institute is to be held at Liverpool from July 14 to July 19.

### A War Memorial.

A memorial to those of the staff of the Third Northern General Hospital who died on service in the war has been unveiled in the Parker transept of Sheffield Cathedral.

### Blackall Gratitude Fund.

The Blackall Gratitude Fund, formed in recognition of the sacrifice of both hands of Mr. Reginald Blackall in consequence of his X-ray work at the London Hospital, reached a total of £1,030.

### A Legacy Declined.

The Herefordshire General Hospital has declined a legacy of £50 conditional upon the hospital board maintaining in good order the graves of the mother, brother and sister of the testatrix. The legal difficulties involved explain the board's decision.

### Tuberculosis Conference.

A full report of the proceedings at the Ninth Annual Conference of the National Association for the Prevention of Tuberculosis, which was held on July 12 and 13 last year, is published by Messrs. George Putnam, of 24-27 Thayer Street, W. 1.

### New X-Ray Block for Halifax Infirmary.

Mr. Joseph Whitaker has offered to build and equip a new X-ray block at Halifax Royal Infirmary. The estimated cost is £6,000. The block will be a memorial to the donor's parents and to his son who was on the medical staff of the infirmary.

### New Treasurer of Guy's Hospital.

Lord Goschen having resigned the Treasurership of Guy's Hospital in consequence of his appointment as Governor of Madras, Mr. Francis Pelham Whitbread has been appointed Treasurer. He has been a governor since 1910, and since 1919 he has been Vice-Chairman of the House Committee.

### Bath Propaganda Scheme.

The Bath and District Friendly Society's Council, which started a hospital propaganda scheme in the autumn, has already distributed some 8,000 collecting-boxes in the wards of the city and the adjoining districts. When 12,000 have been distributed, an income of £5,000 a year is expected from the scheme.

### A Novel Method of Collecting.

A novel method of collecting on behalf of the British Empire Cancer Campaign has been adopted by Mrs. Burman, of Bishop Monkton, Yorks, who is touring the country with a pony only twenty-six inches in height harnessed to a small Ralli car. The latter is stocked with literature dealing with the campaign and decorated with posters and collecting-boxes.

### Beds for Paralysed Working Men.

The committee of Ellerslie House, the home for paralysed soldiers and sailors at Nottingham, has decided to use the beds at present vacant and not likely to be occupied by ex-Service men in future to permanently paralysed working men of Nottingham and Derby on such terms and for so long as the committee might approve.

### Site for Aberdeen Infirmary.

Dr. David Lawson states that in all probability the Aberdeen Infirmary for Sick Children will be presented, through a trust, with the site of 111 acres at Forresterhill, Burnside, that has been purchased for £35,000. This would enable the much-discussed scheme for a joint hospital for Aberdeen to be carried out. The advantages of centralisation and consequent economy are urged.

### Dr. Bengue's Balsam.

A preparation which may be found to give relief in cases of acute rheumatism, gout, neuralgia or neuritis is Dr. Bengue's Balsam, which is a combination of menthol, salicylate of methyl and lanoline. This combination is said to form an absorbent and healing balsam, possessing antiseptic as well as analgesic properties. It is largely used by the medical profession and can be obtained in neat little tubes at 2s. and 3s. Dr. Bengue's Balsam has also been found effective in acute affections of the throat, in cases of hay fever, and in skin troubles.

### The Ideal Health Visitor.

"The Ideal Health Visitor" is the title of a little pamphlet by Lily Skene (John Bale, Sons and Danielson, 6d. net) which emphasizes the moral side of the work. "Health visiting," says the author, "can be as great and inspiring as the Foreign Mission Field, and while we do our official work, what we are will uplift souls to a higher, happier, nobler plane."

### A Marking Ink.

If linen is sent to the laundry unmarked or insufficiently marked, the owner has only himself to blame for its loss. A sixpenny bottle of John Bond's "Crystal Palace" Marking Ink would ensure a clear marking which no amount of washing would impair. With every shilling bottle of this excellent ink, an improved linen stretcher and a special marking pen are given away.

### The Rockefeller Gift to Cambridge.

The Senate of the University of Cambridge have accepted the offer of the Trustees of the Rockefeller Foundation to endow the School of Pathology to the extent of £100,000 for the building and its maintenance, with a further £33,000 towards the School endowment, provided the University can secure the remaining £33,000 necessary for the completion of the endowment.

### Cardiff Royal Infirmary.

Four organisers of a sweep on behalf of the Cardiff Royal Infirmary, and in connection with the Manchester November Handicap have been fined 40s. each under the Lotteries Act. When the race was abandoned, ticket-holders who drew horses shared the prize-money, and the balance went to the hospital to the management committee of which two of the defendants belonged.

### Doctor's Prize for an Essay.

Dr. Alfred Mantle, of Harrogate, has been awarded a prize of £120, open to competition in this and other countries, left by the late Dr. Liddle, a medical officer to the London Hospital Medical College, for the best essay on the cause and prevention of rheumatic fever. In awarding the prize the number and importance of the original observations made by the competitor were to receive special consideration.

### General Nursing Council for Scotland.

The General Nursing Council for Scotland has issued its "Arrangements for Conducting Examinations." Any nurse who has completed three years' training in an approved hospital after November 1, 1919, and before October 1, 1925, may apply for registration, under certain conditions, as an intermediate nurse without passing the examinations. Particulars can be obtained from the Registrar of the Council, 13, Melville Street, Edinburgh.

### For Hospital Laundries.

The authorities of hospitals and other institutions having their own laundries may be glad to have their attention directed to the various laundry machines manufactured by the well-known firm of Messrs. Manlove, Alliott and Co., Ltd., engineers, of Bloomsgrove Works, Nottingham. These include washing-machines, hydro-extractors and ironing-machines for large, medium and small laundries, and possess all the latest improvements in detail and are guaranteed to be thoroughly economical in labour and running costs. A circular giving further particulars can be obtained on application to the firm.

### New President of the League of Mercy.

The King has appointed Princess Mary to be Lady Grand President of the League of Mercy in succession to Princess Alice Countess of Athlone, who resigned on the appointment of the Earl of Athlone to be Governor-General of South Africa. The Prince of Wales (Grand President) has appointed Princess Louise Duchess of Argyll to be Lady President of the Scottish branch of the League. His Royal Highness has also appointed Major Colin MacRae, of Feoirlinn, C.B.E., D.L., to be honorary secretary of the League, and has approved the appointment of Sir William Bull, Bt., M.P., as its honorary solicitor.

### Paripan Enamel.

Under the instructions of the architect Paripan Enamel has been used to a considerable extent in the High Wycombe Memorial Hospital, the opening of which we recorded last month. On some of the corridor walls where enamel was not considered necessary $H_2O$ Water Paint, also manufactured by Paripan, Ltd., was employed. This paint is supplied in white and thirty standard colours. A number of excellent reports from the decorating and building trades have been received by the makers in connection with $H_2O$ Paint, as well as institutions and public offices; and the paint can be applied with good results to ordinary painted or varnished surfaces, and also on Paripan Glossy and Paripan Flat.

### Post-Graduate Lectures for Dublin Nurses.

A series of professional lectures for nurses and midwives has been organised by the Irish Nurses' and Midwives' Union, who have been enabled, through the courtesy of several well-known Dublin doctors, who are giving their services, to arrange the following programme:—"Infant Feeding," Dr. Theobald, Assistant-Master, Rotunda Hospital; "The Elements of Nose, Throat and Ear," Dr. Horace Law; "Gynaecology," Dr. Bethel Solomons; "Insulin," Dr. Henry Moore; "Surgery," Dr. R. Atkinson Stoney; and "Child Welfare," Dr. Alice Barry. The lectures will take place on alternate Thursdays, 5.30—6.30 p.m., in the Nurses' Club, 54, Fitzwilliam Square, Dublin.

### Death Under Anæsthetics.

At a recent inquest in which the jury returned a verdict of "Death accelerated by chloroform, given as a general anæsthetic for a necessary operation," Dr. Waldo, coroner for the City of London and Southwark, drew attention to suggestions made by him and by a Departmental Committee of the Home Office, as far back as 1910, that research work in connection with anæsthetics is urgently needed and could be best done by the appointment of a small standing Scientific Committee nominated by the Home Office and paid out of public funds. It is not sufficient, he said, that instruction in the administration of anæsthetics is an essential part of the medical curriculum; research in connection with anæsthetics alone will prevent the occurrence of these lamentable deaths.

### Nursing of Pulmonary Tuberculosis.

The Society of Superintendents of Tuberculosis Institutions have prepared a scheme to secure a satisfactory standard in the nursing of pulmonary tuberculosis. The scheme incorporates the work demanded by the General Nursing Council for its preliminary examination—the syllabus for this can be obtained from the General Nursing Council—together with special training in the nursing of tuberculous cases, this training being for two years, of which one year will be remitted in the case of nurses who are on the General Register of the General Nursing Council. Particulars of the training may be obtained from the Secretary, Society of Superintendents of Tuberculosis Institutions, Cheshire Joint Sanatorium, Market Drayton, Salop.

### Lectures in Aid of London Hospitals.

The success of the lectures and counter-lectures held last year by public men under a committee of the Hospitals of London Appeal is being followed by a further series of discussions, of which particulars can be gained from the secretary of the London School of Economics, Houghton Street, W.C. 2. The lectures and counter-lectures include: Mr. Pett Ridge and Sir Chartres Biron ("So this is London!"); Miss Rose Macauley and Mr. A. P. Herbert ("What the Public want—and why"); Miss Cicely Hamilton and Sir Edward Marshall Hall, K.C. ("The Play, the Player, and the Playgoer"); Mrs. Belloc Lowndes and Mr. Travers Humphreys ("Is Truth really stranger than fiction"); Lady Frances Balfour and Miss Viola Tree ("Is the Young Woman of the day any worse than she was?"); and Mr. James Agate and Mr. Philip Guedalla ("Hot blood or cold blood?").

### A Successful Commemoration.

Mr. K. A. Macaulay, the President of the West Bromwich and District Hospital, has expressed his satisfaction that the West Bromwich Hospital Saturday Committee, to commemorate their Jubilee Year, 1923, has been able to raise by means of a ballot the money required to provide an anæsthetic room and a room for pathological work, and has promised to give the £400 needed for the erection of a room for storage. These gifts are the outcome of a special report received from the Medical Committee of the hospital, and the Board have placed on record their appreciation of the generosity of the Hospital Saturday Committee and of the President (Mr. Macaulay).

### Nurses in India.

The Annual Home Report of Lady Minto's Indian Nursing Association states that, owing to the unfortunate failure of the Alliance Bank of Simla, Limited, and the consequent low state of finances it was impossible to send out any Sisters in the early part of the year. Owing to the generous support of Lord Inchcape and the P. and O. Company, who have agreed to give £500 for the next three years to meet the travelling expenses, twelve Sisters sailed for India during November and December. One of these had given up an excellent post in England in order to rejoin for a second time, which proves that the Association is becoming popular with the Nursing profession, and there is no longer any difficulty in obtaining candidates for service in India.

### The Best Baby Week Campaign.

The Astor Silver Challenge Shield, which is awarded by Viscount Astor, Chairman of National Baby Week Council, for the most effective baby-week campaign, has been won for 1923 by West Bromwich, and will shortly be publicly presented. An effective campaign was also conducted by the Littlehampton and Arundel Committee, which was awarded a consolation prize, which is to take the form of a banner. The baby-week held in East and West Molesey was remarkable for the large amount of publicity it secured for the maternity and child welfare movement at comparatively little outlay. Dudley secured the fourth place in order of merit; while Leicester health-week, which included a large amount of maternity and child welfare publicity, received honourable mention, though ineligible for the competition, as it had not been announced as a combined health and baby week.

### Extension of St. Mark's Hospital.

The Lord Mayor, presiding at the Mansion House at the eighty-eighth annual general meeting of St. Mark's Hospital, made an appeal for a balance of £12,000 for the extension of the hospital buildings in the City Road. This extension would enable the governors to provide other twenty-one beds—an increase much to be desired in view of the pitiable letters constantly being received from would-be patients anxiously seeking treatment. At present they are sometimes on the waiting list for three months before gaining admission. The proposed extension would also add to the accommodation for out-patients, 1,184 of whom made 3,951 attendances last year. St. Mark's Hospital is practically the only special institution of its kind in the world, and patients come to it from all parts of the globe. Apart from these facilities it has become the Mecca for surgeons throughout the world who wish to study this special branch of surgery. Further information can be obtained from the Secretary, St. Mark's Hospital for Cancer, etc., City Road, by whom much-needed contributions will be gratefully acknowledged.

### Imperial Social Hygiene Congress.

An Imperial Social Hygiene Congress, organised by the National Council for Combating Venereal Diseases, is to be held at the British Empire Exhibition from May 12th to 16th. It will be opened by the Minister of Health, and a review of the existing situation and, it is hoped, a forecast of the future, will be given by the Chief Medical Officer of the Ministry of Health and a medical member of the Scottish Board of Health. The Dominions Overseas and the Crown Colonies have been invited to send representatives, and social hygiene in its national and international aspect will hold a prominent place. Attention will be devoted to the education problem, and, under the Presidency of Viscount Astor, Professor J. A. Thomson and Sir Frederick Mott will define the problem of training the young in positive sex control, stating the biological and sociological reasons for maintaining a monogamous system of marriage and a stable family. "The Place of the Local Authority in the Venereal Diseases Campaign" will be discussed and there will be a conference of special interest to Venereal Disease Officers, to Hospital Almoners, and all concerned with securing the efficiency of the Venereal Disease Clinic.

